# Supplementary material for: Chromothripsis during telomere crisis is independent of NHEJ, and consistent with a replicative origin
Source: Genome Res. 2019 May;29(5):737–49. doi: 10.1101/gr.240705.118 (PMC6499312; doi:10.1101/gr.240705.118)
Supplement: Supplemental Material [file supp_gr.240705.118_Supplemental_file_1.zip › contigs/annotated_contigs/DB57/contig.2.DB57_length_604_mean_cov_5.55629139073.docx]

**DB57_length_604_mean_cov_5.55629139073**

```
TTTGCATATTTATCAGATGTGCCCGCCTTTCTTTTTATTTCATTTTTTTCTTTTTTTCTTTCCTTTTCTCTTCCTTTCCTTTTCTTTCT
 >chr7:145877343-145877610 + E=6e-149
TCCTTTCTTTATCCTTCATTTTTCTTTCTTTTCCTTCCTTTTAATTTATTTTTTAAAGTTGTTAAACTTTAAGATAAAATCAATGGGGC

ATATATCCATTCTCTATCAAAATAGATCTGCCTTATAAATATGTTTCATTTAAAAGTCGATAAATTTTTAAATTCCCAGTATTAATGT|

G|AGAAGAAAACACATTGCAGGAAAAAATCAAACAAATGGCAAAAGGTGGATGAGCGAGACGATCCATTGCATTCCTCTGTCAGCTATC
>chr7:153596278-153596588 - E=8e-166
TCCCCAGCTCTCTTTAGTGCCAATGAATGATTTCCTTCAGCCTGAAATTATGGCTGGATGGTATGATTAGAGGCGACCAAACTGTCCTG

GGAGCTGGGCTCAGCTTCCTCACGTTTCCCAACATAAAGGCAAGGGTAGACATATCACAAACCTGGGTAAGAATACACATCTGGCTTAA

GATAAGCAAAGAAGGAGGAGGAGAAGGAGGAGGAGGAGGAGGAG|GAGAAAGAAGAGGGGGAGAGGGGAGAGG
```
